# GAPDH is not regulated in human glioblastoma under hypoxic conditions

**DOI:** 10.1186/1471-2199-8-55

**Published:** 2007-06-27

**Authors:** Harun M Said, Carsten Hagemann, Jelena Stojic, Beate Schoemig, Giles H Vince, Michael Flentje, Klaus Roosen, Dirk Vordermark

**Affiliations:** 1 University of Würzburg, Dept. of Radiation Oncology, Germany; 2 University of Würzburg, Dept. of Neurosurgery, Tumorbiology Laboratory, Germany

## Abstract

**Background:**

Gene expression studies related to cancer diagnosis and treatment are becoming more important. Housekeeping genes that are absolutely reliable are essential for these studies to normalize gene expression. An incorrect choice of housekeeping genes leads to interpretation errors of experimental results including evaluation and quantification of pathological gene expression. Here, we examined (a) the degree of regulation of GAPDH expression in human glioblastoma cells under hypoxic conditions *in vitro *in comparison to other housekeeping genes like β-actin, serving as experimental loading controls, (b) the potential use of GAPDH as a target for tumor therapeutic approaches and (c) differences in GAPDH expression between low-grade astrocytomas and glioblastomas, for which modest and severe hypoxia, respectively, have been previously demonstrated. GAPDH and β-actin expression was comparatively examined *in vivo *in human low-grade astrocytoma and glioblastoma on both protein and mRNA level, by Western blot and semiquantitative RT-PCR, respectively. Furthermore, the same proteins were determined *in vitro *in U373, U251 and GaMG human glioblastoma cells using the same methods. HIF-1α protein regulation under hypoxia was also determined on mRNA level *in vitro *in GaMG and on protein level in U251, U373 and GaMG cells.

**Results:**

We observed no hypoxia-induced regulatory effect on GAPDH expression in the three glioblastoma cell lines studied *in vitro*. In addition, GAPDH expression was similar in patient tumor samples of low-grade astrocytoma and glioblastoma, suggesting a lack of hypoxic regulation *in vivo*.

**Conclusion:**

GAPDH represents an optimal choice of a housekeeping gene and/or loading control to determine the expression of hypoxia induced genes at least in glioblastoma. Because of the lack of GAPDH regulation under hypoxia, this gene is not an attractive target for tumor therapeutic approaches in human glioblastoma.

## Background

The appropriate choice of an internal standard is critical for quantitative protein and RNA analyses. Glyceraldehyde-3-phosphate dehydrogenase (GAPDH) is a glycolytic enzyme that possesses diverse functions that are independent of its role in glycolysis [1]. GAPDH is a multifunctional enzyme overexpressed in many tumors and induced by hypoxia in normal and malignant cells. The degree to which hypoxia transcriptionally activates GAPDH is cell- type specific [2].

GAPDH is considered to be a "housekeeping gene". Previous contributions showed that GAPDH expression is regulated by a variety of factors like calcium [3], insulin [4], and hypoxia [5], although the transcription factor hypoxia-inducible factor-1 (HIF-1) regulates the expression of genes which are involved in glucose supply, growth, metabolism, redox reactions and blood supply. The HIF family comprises the HIF-1α, HIF-1β, HIF-2α, and HIF-3α subunits [6]. Under normoxic conditions, the HIF-1α subunit is undetectable because it undergoes rapid ubiquitination and proteosomal degradation [7,8].

Hypoxia is characterized by inadequate oxygen delivery to the tissue with a resulting imbalance between oxygen demand and energy supply [9]. As a consequence, HIF-1-regulated hypoxia-induced genes are transcribed [10-15]. Many of the proteins encoded by these genes are involved in adaptive responses counteracting a detrimental impact of hypoxia, including erythropoiesis, angiogenesis, iron homeostasis, glucose and energy metabolism, as well as cell proliferation and survival decisions [10].

There are two types of hypoxia: transient and chronic hypoxia. Transient hypoxia is a temporary reduction in oxygen availability. The inadequate vascular geometry relative to the volume of oxygen-consuming tumor cells creates diffusion-limited O_2 _delivery, which results in chronic hypoxia [16,17]. Cells in the hypoxic environment shift from aerobic citric acid cycle (TCA cycle) to anaerobic metabolism (glycolysis, also known as Warburg effect), as a consequence to chronic hypoxic conditions. The response to low O_2 _levels is given by up-regulating the synthesis of HIF [6]. Tumors typically contain hypoxic regions, since tumor vasculature is dysfunctional and unable to meet the metabolic needs of rapidly proliferating cancer cells [18]. Tumor cells are resistant to therapeutic approaches, like ionizing radiation and chemotherapy. For ionizing radiation the dose required to produce the same amount of cell killing is up to three times higher for hypoxic cells than for well-oxygenated cells [19]. In glioblastoma, the modification of tumor oxygenation and thus radiosensitivity is an attractive approach to improve the prognosis of glioblastoma patients currently tested in clinical trials [20].

In previous work, it has been shown that GAPDH expression increases as a repose to the hypoxic development in endothelial cells [5,21-23]. Regulation of GAPDH by hypoxia appears to be cell- type specific. For instance, GAPDH expression is induced to a much lesser extent in fibroblasts and smooth muscle cells than it is in endothelial cells [24].

In the present study we addressed the question whether GAPDH expression is regulated by hypoxia in human glioblastoma cells *in vitro *and in human glioma tumor samples. The answer of this question will provide insight into practical applications of GAPDH as an internal standard in investigations of hypoxia-inducible gene expression or as a target for tumor therapeutic approaches in human glioblastoma. We also aimed to determine the validity of two further housekeeping genes for their use as internal standards in experimental cancer research.

## Results

### GAPDH mRNA expression is not regulated by hypoxia, neither *in vivo *in human glioma tissue, nor *in vitro *in human glioblastoma cell lines

**Figure 1:**
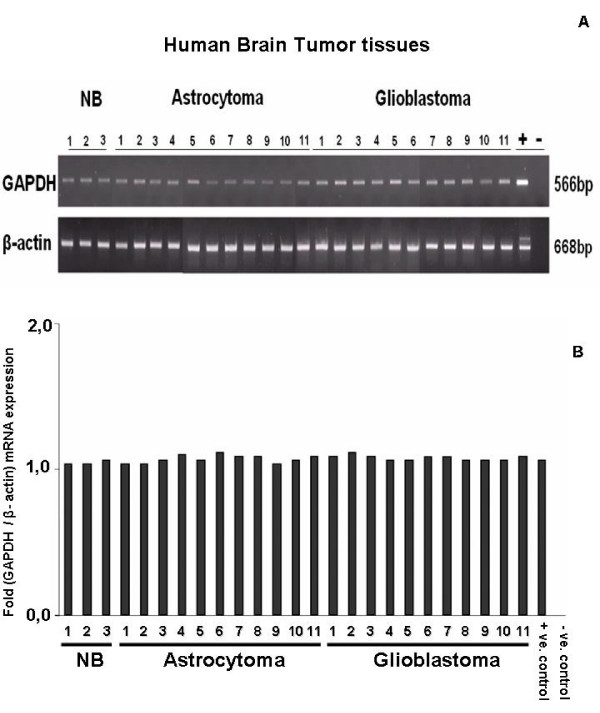
***In vivo *mRNA expression of human housekeeping genes GAPDH and β-actin in biopsies of normal brain (NB), low-grade astrocytoma and glioblastoma. (A)**. Semiquantitative RT-PCR screening of tissue lysates from three normal brain, 11 astrocytoma and 11 glioblastoma samples. GAPDH and β-actin mRNA is homogenously expressed in all analysed samples. + = positive control, - = negative control using water as template. (B) Expression intensities of the PCR-bands were densitometrically evaluated. The bar graphs show GAPDH expression after normalization to the corresponding expression of β-actin. GAPDH was not regulated by the severely hypoxic tumor environment typical of glioblastoma.

Both GAPDH and β-actin mRNA was expressed in 22/22 brain tumor samples (11 low-grade astrocytoma and 11 glioblastoma) and in 3/3 normal brain tissue samples, as shown by semiquantitative RT-PCR (Fig. 1A). Densitometric analysis did not show any differences in GAPDH mRNA expression between the two tumor types with different levels of tumor oxygenation (Fig. 1B). Each value was normalized to the corresponding expression of the housekeeping gene β-actin.

**Figure 2:**
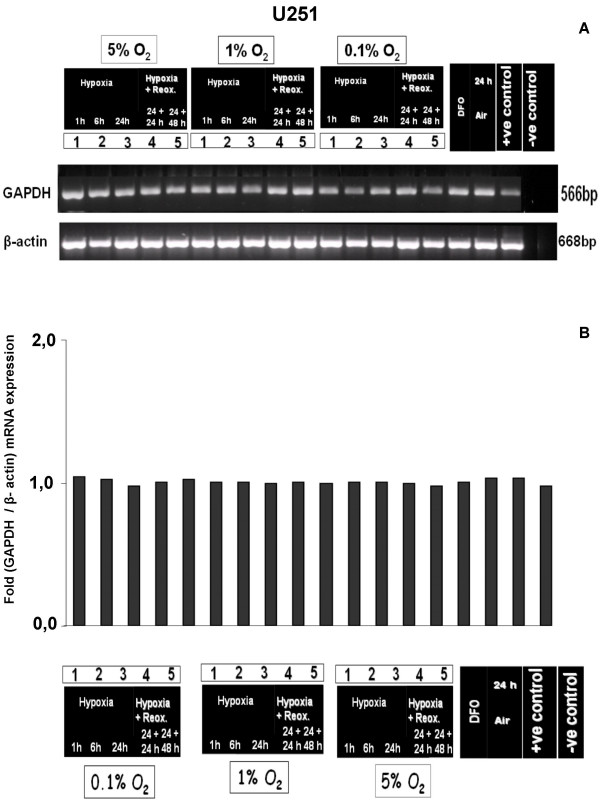
**Effect of different hypoxic conditions on GAPDH mRNA expression in U251 cells**. Cells were cultured 24 h under normoxic conditions or for 1 h, 6 h or 24 h under hypoxic (0.1%, 1%, 5% O_2_) conditions. Reoxygenation experiments were performed for 24 h and 48 h after 24 h hypoxia. DFO served as hypoxia-positive control. **(A) **Semiquantitative RT-PCR analysis of GAPDH and β-actin mRNA expression. Shown is one representative experiment out of three. **(B) **Bar graphs showing GAPDH expression strength after densitometric evaluation of the PCR bands and normalization to the corresponding expression of β-actin. GAPDH mRNA expression was not regulated by hypoxic conditions.

**Figure 3:**
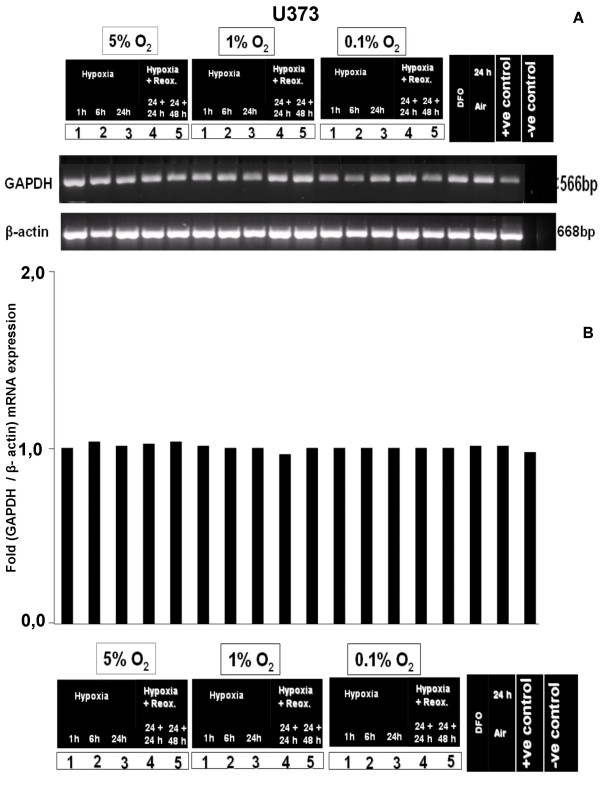
**Effect of different hypoxic conditions on GAPDH mRNA expression in U373 cells**. For experimental settings refer to Fig. 2. **(A) **Semiquantitative RT-PCR analysis **(B) **densitometric evaluation of GAPDH and β-actin mRNA expression.

To investigate the effect of controlled hypoxic conditions on GAPDH mRNA expression, we performed *in-vitro *cell culture assays with 5%, 1% and 0.1% O_2 _with and without reoxygenation. No regulatory effect of these different oxygenation conditions on GAPDH expression was detectable by semiquantitative RT-PCR in the human glioblastoma cell lines U251, U373 and GaMG (Fig. 2A, 3A, 4A). The densitometric evaluation confirmed these results (Fig. 2B, 3B, 4B). Together, these data suggest that the known formation of hypoxic regions within gliobastoma tumor tissue is not accompanied by an upregulation of GAPDH mRNA.

**Figure 4:**
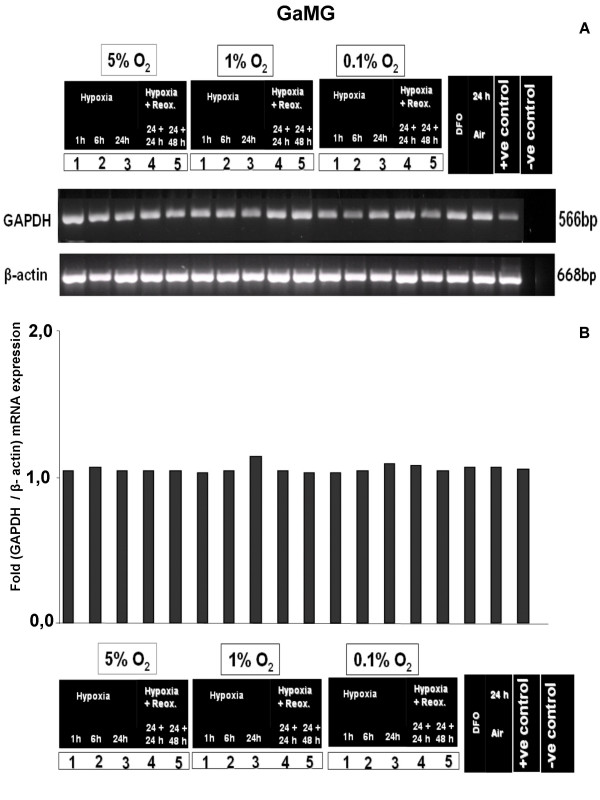
**Effect of different hypoxic conditions on GAPDH mRNA expression in GaMG cells**. For experimental settings refer to Fig. 2. **(A) **Semiquantitative RT-PCR analysis **(B) **densitometric evaluation of GAPDH and β-actin mRNA expression.

### Hypoxic conditions do not influence GAPDH protein expression *in vivo *in human brain tumor samples or *in vitro *in glioblastoma cell lines

**Figure 5:**
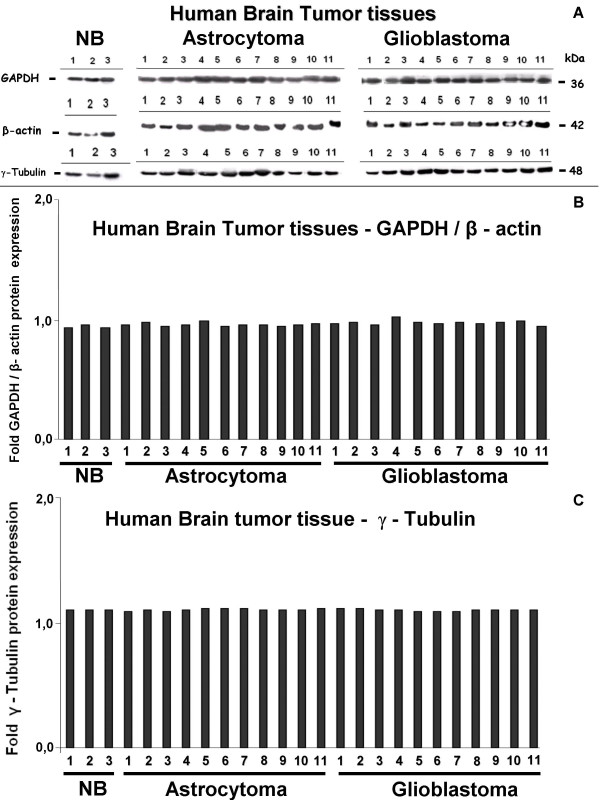
***In-vivo *protein expression of human housekeeping genes GAPDH, β-actin and γ-tubulin in biopsies of normal brain (NB), low-grade astrocytoma and glioblastoma. (A) **Western-blot analysis of tissue lysates. GAPDH, β-actin and γ-tubulin are homogenously expressed by all analysed samples. (B) Expression intensities of the bands in the Western blot were densitometrically evaluated. The bar graphs show GAPDH expression after normalization to the corresponding expression of β-actin. No GAPDH upregulation in the more hypoxic glioblastoma samples in comparison to low-grade astrocytoma was detectable. (C) Densitometric evaluation of γ-tubulin expression in the human brain tumor tissues. Band intensities are shown as bar graphs. No regulatory effect of hypoxic conditions *in vivo *on housekeeping gene expression could be detected.

To exclude translational regulation of GAPDH protein expression by hypoxic conditions, Western-blot analysis was performed using lysates from the same tumor samples described above. Again, GAPDH, β-actin and as an additional housekeeping gene γ-tubulin was detected in all samples analyzed (Fig. 5A). Expression of all three proteins was very homogenously distributed among the two tumor entities, low-grade astrocytoma with known modest tumor hypoxia and glioblastoma with known severe hypoxia (densitometric analysis, Fig. 5B and 5C).

**Figure 6:**
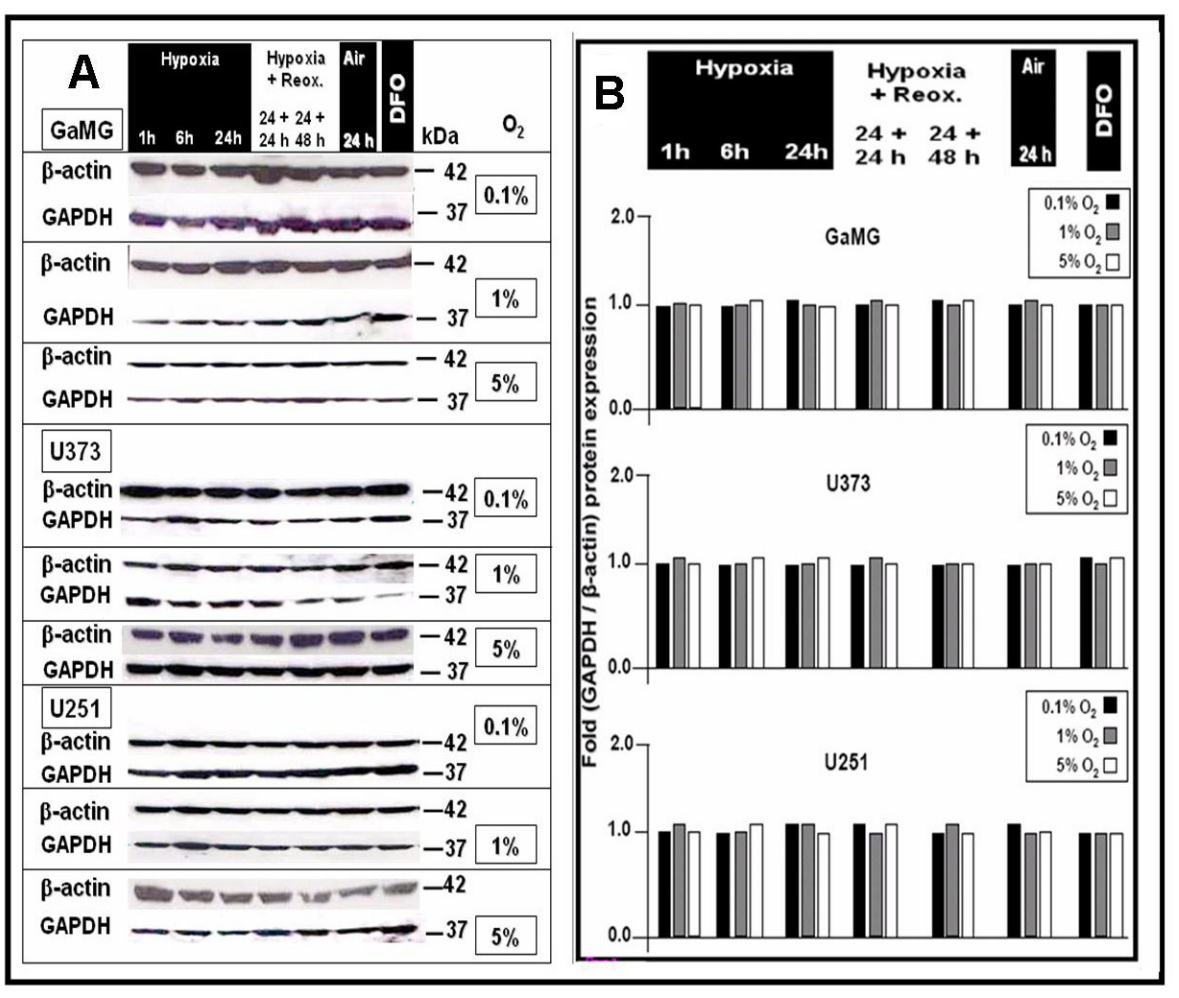
**Effect of different hypoxic conditions on GAPDH protein expression in GaMG, U373 and U251 cells**. For experimental settings refer to Fig. 2. **(A) **Western-blot analysis of β-actin and GAPDH protein expression. Shown is a representative experiment out of three. **(B) **Bar graphs showing expression strength of GAPDH after densitometric evaluation of signal strengths and normalization to the corresponding β-actin expression. Hypoxia did not regulate GAPDH expression in any direction.

Glioblastoma cell lines GaMG, U373 and U251, which were cultured under different hypoxic conditions as described above, did not show any regulation of GAPDH protein expression (Fig. 6A and 6B, respectively). Therefore, GAPDH mRNA and protein expression is not modified in response to different oxygenation, hypoxia or reoxygenation conditions *in vitro *in the tested cell lines and not differently expressed in human tumor glioma samples with known different levels of hypoxia.

### HIF-1α regulation in response to hypoxia

**Figure 7:**
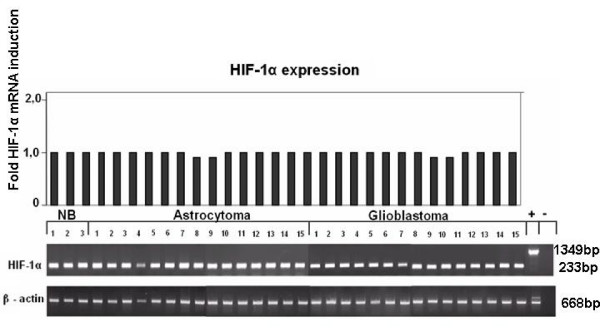
**HIF-1α mRNA expression *in vivo *in different human brain tissues, detected by semi-quantitative RT- PCR**. HIF-1α mRNA expression was comparable in normal brain (NB), low-grade astrocytoma and glioblastoma. + = positive control using genomic DNA as template, - = negative control with water as template. The bar graphs show band intensities after densitometric evaluation and normalization of HIF-1α expression to the corresponding β-actin expression.

Semiquantitative RT-PCR revealed that HIF-1α is evenly expressed in normal brain and astrocytic tumor tissues and that there is no upregulation of HIF-1α mRNA in glioblastoma samples in comparison to low-grade astrocytomas (Fig. 7).

**Figure 8:**
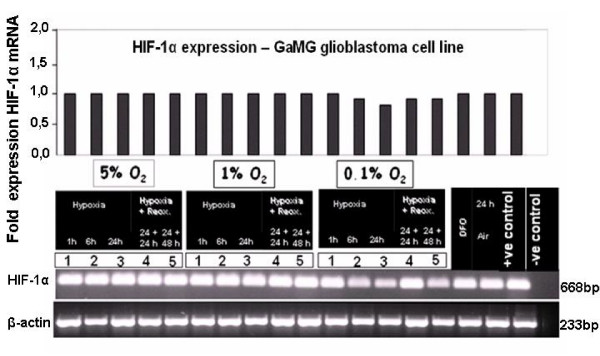
**HIF-1α mRNA expression in human GaMG glioblastoma cell line after *in vitro *application of different hypoxic conditions**. For experimental settings refer to Fig. 2. Semiquantitative RT-PCR did not reveal any regulatory event under different oxygenation, hypoxia and reoxygenation conditions. Bar graphs show band intensities after densitometric evaluation and normalisation to β-actin expression as it is known from previous experiments. Representative experiment out of three.

**Figure 9:**
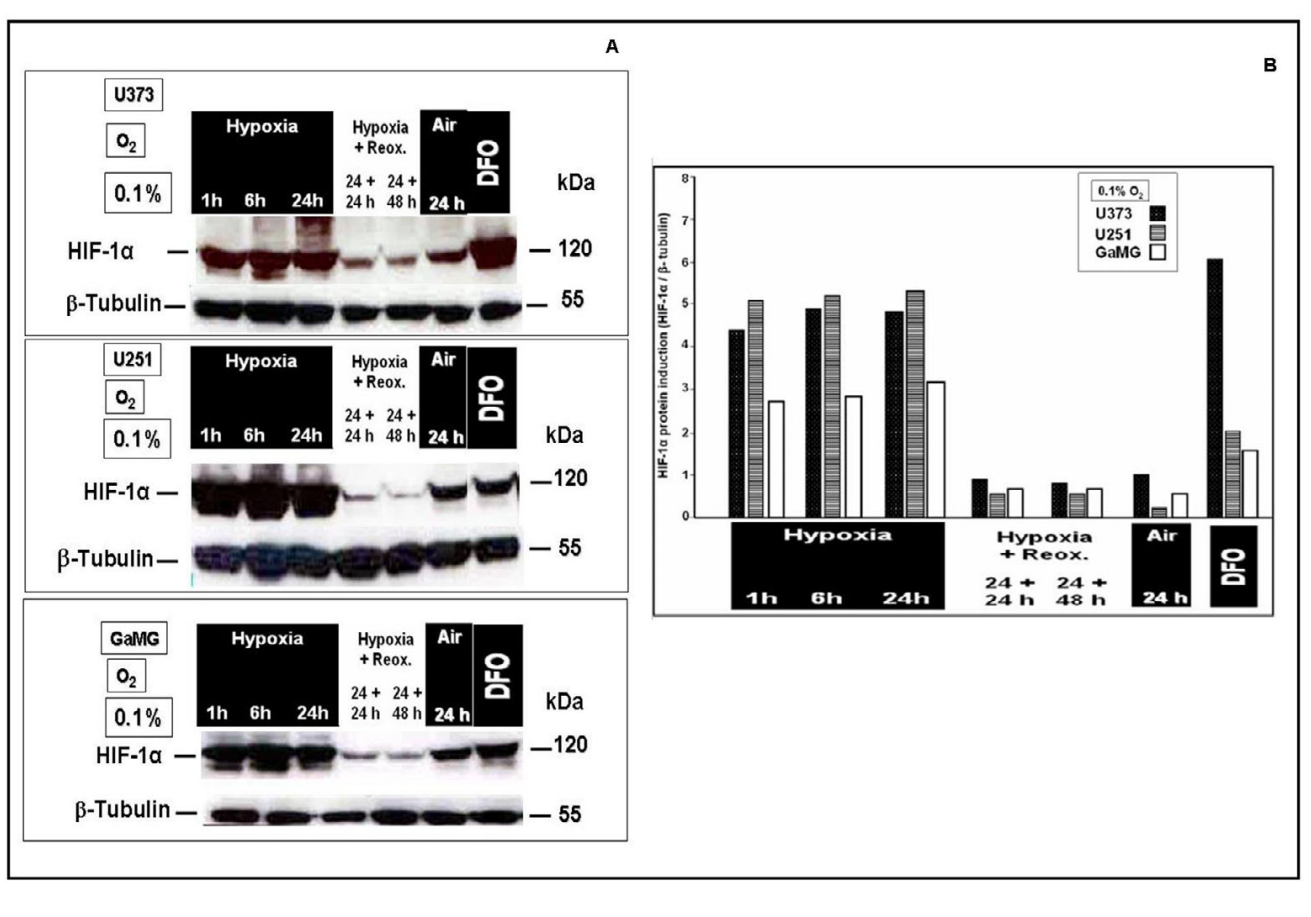
**Nuclear protein expression of HIF-1α in human U373, U251 and GaMG glioblastoma cells after *in vitro *application of different hypoxic conditions**. For experimental settings refer to Fig. 2. (A) Western-blots. β-tubulin served as loading control. (B) HIF-1α protein expression strength shown as bar graphs after densitometric evaluation and normalization to the corresponding β-tubulin expression. HIF-1α was strongly expressed after 1 h at 0.1% O_2_, and still increased for up to 24 h hypoxia. It showed stable reduced expression after up to 48 h reoxygenation. Similar data were obtained in three independent experiments.

In a representative *in-vitro *example in GaMG cells cultured under different oxygenation conditions, HIF-1α mRNA expression was not influenced by hypoxic conditions (Fig. 8). In contrast, HIF-1α nuclear protein clearly responded with upregulation under hypoxic and downregulation under reoxygenation or normoxic conditions in U373, U251 and GaMG (Fig. 9). HIF-1α was strongly expressed after 1 h at 0.1% O_2_, still increased after 24 h hypoxia and showed stable reduced expression upon reoxygenation up to 48 h (Fig. 9).

These results confirm oxygen-dependent regulation of HIF-1α at the protein level in the experimental models of the present study and reassure that the experimental settings for expression analysis of GAPDH were suitable to evaluate regulatory events by hypoxic conditions.

## Discussion

It has been postulated that GAPDH protein expression is regulated as a consequence of the hypoxic development of the cellular environment *in vitro *[5,21-26]. Several authors showed in their models that GAPDH mRNA expression was regulated during hypoxic events. Some also presented that the application of 18S-, 28S-RNA or β-actin instead as a loading control for experiments involving reduced oxygen concentration is more suitable for this purpose than GAPDH. Housekeeping genes are normally present in all cells and their expression levels should remain relatively constant under different experimental conditions. It is logic that no single housekeeping gene always possesses stable expression levels under all experimental conditions. Therefore, it is necessary to characterize the suitability of various housekeeping genes to serve as internal RNA controls under particular experimental conditions where transcription effects are being tested.

To exclude a potential influence of oxygen concentrations on GAPDH expression as a confounding factor we have previously employed an additional control, 18S RNA, in experiments of hypoxia-inducible gene expression [21]. In these experiments, expression of GAPDH under different oxygen concentrations (severe hypoxia, normoxia and reoxygenation), was compared to the 18S RNA detected. They showed that GAPDH was not significantly regulated under hypoxic conditions in a panel of human tumor cell lines *in vitro*, and the expression of the gene examined was not altered after substitution of the GAPDH by the 18S RNA band with subsequent densitometric evaluation [21].

GAPDH induction by hypoxia in endothelial cells occurs via mechanisms other than those involved in other hypoxia-responsive systems [24]. A lack of regulation of GAPDH mRNA in response towards hypoxic events has also previously been demonstrated in the case of articular chondrocytes [28]. Table 1 summarizes literature data about GAPDH expression in response to the hypoxic development of the cellular environment by several tumor and non-tumor cells.

**Table 1:** Overview of GAPDH expression by different tumor and non-tumor cell lines as a consequence of the development of a hypoxic cellular microenvironment

**Cell line or type**	**Origin**	**Genetic mutations (-/+)**	**GAPDH overexpression under hypoxia**	**Ref. GAPDH**	**Ref. mutations**
**LNCap**	- Human prostate adenocarcinoma cells	- No mutations	+	[35]	[27]
**ATII**	-Rat alveolar epithelial cells	- No mutations	+	[26]	[26]
**SiHA**	-Human spontaneous cervical cancer cells	- No mutations Wild type p53	+	[36]	[37]
**MBEC4**	-Mouse brain capillary endothelial cells	- No mutations	+	[38]	[38]
**EC**	- Human endothelial cells	- Not determined Mutated epithelial cells are present	+	[5], [22], [23], [24] [39], [40]	[40]
**Skeletal muscles from hindlimbs of newborn white New Zealand rabbits**	- Rabitts skeletal muscle cells	- Not determined	+	[41]	-
**Bovine articular chondrocytes**	-Bovine chondrocytes	- Not determined	-	[38]	-
**U373 – MG**	-Human malignant glioma cells	-Apoptosis resistant mutant P53 - Peroxisome proliferator activated receptor – γ - PTEN mutation	-	-	[42], [43], [44],
**GaMG**	-Human malignant glioma cells	- No mutations	-	-	[34], [44]
**U251**	-Human malignant glioma cells	-Mutated p53 – P14ARF/P16 deletion	-	-	[43], [46]

Our data did not reveal any correlation between hypoxia induced HIF-1α protein overexpression and GAPDH regulation on mRNA and protein level *in vitro *in human glioblastoma cell lines. Although we did not measure oxygenation levels directly in the human tumors, samples of which were analyzed regarding GAPDH expression, we considered low-grade astrocytoma and glioblastoma as tumor entities characterized by modest hypoxia and severe hypoxia, respectively. This has been suggested by experimental findings of needle electrode measurements of human glioma [29] and by immunohistochemical studies using HIF-1α or carbonic anhydrase IX (CA IX) as an endogenous hypoxia marker [30,31] or EF5 as an injectable hypoxia marker [32]. Furthermore, our own recent studies showed significant mRNA overexpression of known hypoxia-regulated genes in glioblastoma, as compared to low-grade astrocytoma [33]. Based on these findings from other studies, our present results suggest that there is also no hypoxia-dependent regulation of GAPDH in astrocytic tumors *in vivo*.

## Conclusion

Therefore, we can conclude that the regulation of GAPDH mRNA and protein expression as a response to the hypoxic development in the tumor cell enviroment *in vitro *and *in vivo *is not an absolute phenomenon, but occurs as a cell-specific post-transcriptionally regulated event. Expression of GAPDH represents one of the alternatives of a housekeeping gene and can be used as a loading control in experiments with glioma cells. Therapeutic strategies for treatment of human astrocytic tumors involving GAPDH as target molecule do not represent a valid approach in conjunction with tumor hypoxia in the human brain.

## Methods

### Cell and culture and hypoxia treatment

Early-passage human malignant glioma cell lines U251 and U373 from the American Type Culture Collection (ATCC, Rockville, MD) and GaMG, a cell line that was established from a patient with glioblastoma multiforme (Gade Institut of the University Bergen, Norway) [34], were grown on glass Petri dishes in Dulbecco's modified Eagle's medium, supplemented with 10% fetal bovine serum (FBS) and non-essential amino acids. Additionally, all culture media were supplemented with penicillin (100 IU/ml)/streptomycin (100 μg/ml) and 2 mM L-glutamine. Cells were treated with *in-vitro *hypoxia for 1, 6 or 24 hours at 0.1%, 1% and 5% O_2_, respectively, in a Ruskinn (Cincinnatti, OH, USA) Invivo_2 _hypoxic workstation as previously described [29]. For reoxygenation experiments, dishes were returned to the incubator after 24-hour hypoxia treatment.

### Acquisition of human tumor tissues

Tissue biopsies were obtained surgically from two groups of patients: 11 patients with glioblastoma multiforme and 11 patients with low-grade astrocytoma. Samples were immediately frozen at -80°C and then stored in liquid nitrogen before further analysis. Three samples of temporal brain tissue (normal brain, NB) were a kind gift of Thomas Freiman (University Hospital Freiburg im Breisgau, Germany) and derived from patients with epilepsy. The experimental protocols were approved by the Human Ethics Committee of Würzburg University and were performed according to the Declaration of Helsinki.

### Intracellular β-actin, GAPDH and HIF-1α levels in response to in vitro hypoxia

Intracellular β-actin, GAPDH and HIf-1α levels were detected via immunoblotting of protein lysates and nuclear extracts, or by semiquantitative RT-PCR where β-actin and γ-tubulin served as loading controls, respectively.

### Preparation of nuclear extracts

According to the protocol, 1 × 10^6 ^cells/ml were seeded and at the end of the respective treatment, subconfluent cells were scratched from petri dishes with 10 ml PBS. A pellet was obtained by centrifugation (Beckman CS-6R, 4 min, 800 rpm). After two PBS washes, cells were resuspended in 1 ml PBS and centrifuged at 14,000 rpm for 45 sec. The cell pellet was resuspended in 400 μl ice-cold buffer A (10 mM Hepes, pH 7.9, 10 mM KCl, 0.1 mM EDTA, 0.1 mM EGTA, 1 mM PMSF, 10 μl complete protease inhibitor cocktail (Roche), 1 mM DTT and incubated on ice for 15 minutes. For cell lysis 25 μl of 10% NP-40 was added and cells were homogenized with 10 strokes in a Dounce homogenizer at 4°C, followed by centrifugation for 1 min at 14,000 rpm for nuclei sedimentation. Supernatants were carefully removed and regarded as cytoplasmic fractions. Extraction of nuclear proteins was achieved by adding 50 μl of buffer C (20 mM HEPES [pH 7.9], 0.4 M NaCl, 1 mM EDTA, 1 mM EGTA, 1 mM PMSF, 0.1 μl complete protease inhibitor cocktail).

### Preparation of lysates from cells and human tumor tissues and immunoblotting

Whole-cell lysates were prepared with 0.1 ml RIPA buffer (1 × TBS, 1% Nonidet P-40 (Amresco, Vienna, Austria), 0.5% sodium deoxycholate, 0.1% SDS; protease inhibitors pepstatin A 1.4 μM, aprotinin 0.15 μM and leupeptin 2.3 μM and 100 μM PMSF (all from Sigma, St. Louis, MO, USA). To inhibit protein dephosphorylation, phosphatase inhibitor mix (Sigma) was added. Using a syringe fitted with a 21 gauge needle to shear DNA, lysates were transferred to a microcentrifuge tube, followed by 30 min incubation on ice. Subsequently, cell lysates were cleared by centrifugation at 15.000 × g for 12 min at 4°C. 20 μg of protein lysates were separated by 8% SDS polyacrylamide gel electrophoresis and transferred to a 0.45 μm nitrocellulose membrane (Protran BA 85; Schleicher & Schuell, Dassel, Germany). Nonspecific binding was blocked by 5% non-fat milk powder in TBS overnight at 4°C followed by incubation with the HIF-1α antibody (BD Biosciences, dilution 1:500) with nuclear extracts in 2.5% nonfat milk powder in TBS for 1 h at room temperature. Blots were washed two times in TBS/0.05% Tween-20 (Bio-Rad, Munich, Germany) and subsequently three times in TBS for 5–10 min each. The secondary antibody, in all cases a goat anti-mouse Immunoglobulin/HRP, dilution 1:2000 (P0447 stock solution: 400 μg/ml; DakoCytomation, Denmark), was incubated for one additional hour at room temperature followed by five washes as described above. Bound antibodies were detected by developing the membrane with ECL Plus Western Blotting detection system (Amersham Biosciences, Cambridge, UK) for 5 min with subsequent development of the Hyperfilm ECL (Amersham). Membranes were also probed with anti-β-actin antibody (A 5316, 1:10000, Sigma-Aldrich, Germany), anti GAPDH antibody (Abcam, Ab8245, 1:2000) or anti-β-tubulin mouse monoclonal antibody (Sigma, 1:2000). For reprobing, membranes were stripped with stripping buffer (100 mM β-mercaptoethanol, 2% sodium dodecyl sulfate, 62.5 mM Tris HCl pH 6.7) at 60°C for 30 min.

### Isolation of total RNA from tumor cell lines and tumor tissues

Total RNA was isolated from cultured tumor cells as reported previously [6] and described in [3], including the digestion of contaminating DNA with the provided DNase. Total RNA from tumor tissues was isolated with the nucleospin RNA II kit (Macherey & Nagel, Dueren, Germany) following the manufacturer's instructions.

### Comparison of HIF-1α, β-actin and GAPDH mRNA expression levels in human glioma tissue and human glioblastoma cell lines by semiquantitative reverse transcription-polymerase chain reaction (RT-PCR)

To compare the expression of the individual genes examined, RT-PCR was performed using primers designed using published information on GAPDH, β-actin and HIF-1α mRNA sequences in GenBank (accession numbers NM_002046 for GAPDH, NM_001101 for β-actin and NM_001530.2 for HIF-1α, respectively). An aliquot of 1–5 μg of total mRNA from human gliblastoma and astrocytoma tissue or glioblastoma cell lines was transcribed at 42°C for 1 h in a 20 μl reaction mixture using 200 U RevertAid™ M-MuLV Reverse Transcriptase (RT), oligo(dT)18 primer and 40 U Ribonuclease inhibitor (all from Fermentas, Ontario, Canada).

For PCR-reactions primers were designed in flanking exons with Primer3 software (available online ): to produce an 566 bp amplification product of GAPDH, the forward primer (F1) was 5'-GCAGGGGGGAGCCAAAAGGG-3' (nucleotides 393 – 412) and the reverse primer (R1) 5'-TGCCAGCCCCAGCGTCAAAG-3' (nucleotides 939 – 958). To produce an 668 bp amplification product of β-actin, the forward primer (F1) was 5'-CGTGCGTGACATTAAGGAGA-3' (nucleotides 697 – 716) and the reverse primer (R1) 5'-CACCTTCACCGTTCCAGTTT-3' (nucleotides 1345 – 1364) and to produce an 233 bp amplification product of HIF-1α, the forward primer (F1) was 5'-TTACAGCAGCCAGACGATCA -3' (nucleotides 2516 – 2535) and the reverse primer (R1) 5'- CCCTGCAGTAGGTTTCTGCT -3' (nucleotides 2729 – 2748). The PCR was performed with 25 to 32 cycles with increments of 5 cycles using PCR systems and reagents acquired from Promega™ (Promega GmbH, Mannheim, Germany) and applied according to the manufacturer's instructions. The PCR products were separated on 1% agarose gels (Sigma-Aldrich, Steinheim, Germany) and visualized by ethidium bromide staining (0.07 μg/ml ethidium-bromide; Biorad, Munich, Germany).

### Densitometric evaluation

Densitometric evaluation of signal strengths in Western blots or in semiquatitative RT-PCR was performed with 1D Kodak Image Analysis Software. The amount of DNA or proteins gave signals that were measured in Kodak light units (KLU) and divided by the corresponding signals of the loading control (γ-tubulin, β-tubulin and β-actin for Western blots and semiquatitative RT-PCR) as previously described [11,21].

## Abbreviations

tumor hypoxia, GAPDH, glyceraldehyde-3-phosphate dehydrogenase, β-actin, oxygen, glioblastoma multiforme, astrocytoma, HIF-1α, 18S RNA

## Authors' contributions

HMS was the primary author of the manuscript, performed the *in-vitro *hypoxia experiments, supplied the *in-vitro *mRNA, protein lysates and nuclear extracts, performed the Western blots, densitometric analysis of the results and participated in the study design. CH co- authored the manuscript and supplied the brain tumor samples and their mRNA and protein and participated in the study design. Both HMS and CH also coordinated the group and contributed to the development of the experimental strategy. JS designed the primers used for RT-PCR and participated with BS in the experimental procedures. GHV, MF, KR and DV also participated in the study design. All authors read and approved the final manuscript.
